# Surface water quality impacts from organic versus conventional agricultural systems

**DOI:** 10.1002/jeq2.70208

**Published:** 2026-06-30

**Authors:** Raven Bier, Melinda Daniels, Diana Oviedo‐Vargas, Marc Peipoch, Emma Kelsick, Jinjun Kan

**Affiliations:** ^1^ Savannah River Ecology Laboratory University of Georgia Aiken South Carolina USA; ^2^ Odum School of Ecology University of Georgia Athens Georgia USA; ^3^ Stroud Water Research Center Avondale Pennsylvania USA

## Abstract

Agriculture is integrally intertwined with water, yet we have little information about how the different approaches to growing food influence water quality. Food production through different strategies is driven by economic and environmental concerns. Organic agriculture has increased for several decades and is projected to continue. Here, we conducted a literature synthesis of paired field studies documenting water quality differences from conventional and organic agriculture of terrestrial row‐crops. Using the 37 papers that met our criteria, we compiled water quality parameters and calculated the percentage change between conventional and organic treatment paired comparisons. This synthesis revealed that 79% of the 14 nutrient, soil loss, and runoff parameters with at least five paired comparisons more often showed either improvement or no difference with organic. Specifically, nitrogen and overall soil losses were reduced in organic systems; however, soil loss outcomes were strongly influenced by tillage practices, and fertilizer type could change whether an organic field experienced greater or lesser nitrogen losses. Losses of phosphorus and carbon generally increased under organic agriculture, except for total phosphorus in runoff. Paired‐field trial comparisons were rare in the literature and highlighted the need for additional studies, consistency in the parameters measured, and sufficient data collected to account for variation in fertilizers, tillage intensity, soil types, and topography, among other parameters. As publications comparing conventional and organic soil quality increase rapidly, studies of water quality lag, leaving open the potential for a shift in agricultural approaches to have unintended consequences for this essential resource.

## Core Ideas

- There are more cases of lower nitrogen or soil aqueous export under organic agriculture.
- There are more cases of reduced phosphorus or carbon aqueous export under conventional agriculture.
- Uncertainty remains in the water quality effects from different agricultural managements.

## Plain Language Summary

Agriculture is essential for food production, yet fields can export nutrients, soil, and pesticides that degrade freshwater resources. The two major systems of agriculture, conventional and organic, vary in the practices used to grow food and, thus, may have different effects on water quality. The extent of land used for organic agriculture is small, but growing rapidly. We synthesized studies that compare the water quality effects of side‐by‐side conventional and organic agriculture row‐crop fields. We identified only 37 studies that met the criteria. There were more cases where water leaving organic agriculture fields contained less nitrogen or soil, and more cases where water leaving conventional fields had less phosphorus or carbon. This review highlights the need for more studies from different agriculture systems and regions and shows that individual practices not exclusive to either type of agriculture, such as soil tillage, may be as influential as the category of system.

**Abbreviations**
eC.F.R.: electronic Code of Federal Regulations
LCA: life cycle assessment
S.E.: standard error from the mean
TKN: total Kjeldahl nitrogen
TN: total nitrogen
TP: total phosphorus
TSS: total suspended solids

## 1 INTRODUCTION

Agriculture has long been recognized as the dominant non‐point source of pollution to freshwater ecosystems in high income countries (Carpenter et al., 1998; USEPA, 2016) and is increasingly concerning in low‐income countries (Evans et al., 2019; Landrigan et al., 2018). Many factors drive water pollution from agriculture, including demand for products, intensification of methods, and shifts in farming systems (Mateo‐Sagasta et al., 2018) that are often responsive to broader social, environmental, and economic pressures (Archer et al., 2008; Piñeiro et al., 2020). Managing agricultural pollution and minimizing risk to freshwater resources requires an understanding of how different farming systems alter pollution delivery to aquatic environments.

The pressure to reduce agricultural pollution has coincided with increased consumer demand for organic food, resulting in the rapid expansion of land under organic production. Farmers are responding to organic crop price premiums and reduced production costs (Cavigelli et al., 2008; Delate et al., 2015), as well as to a range of complex personal motivations (Han et al., 2021). As they become more widely adopted, shifts in field‐scale land management can eventually produce watershed‐scale consequences for water quality in receiving ecosystems. While many robust studies have investigated organic farming system impacts on physical, chemical, and biological soil health properties (Christel et al., 2021; Gattinger et al., 2012), comparatively few studies have investigated their influences on surface runoff water quality. Several meta‐analyses have included water quality when comparing organic and conventional agriculture consequences (Mondelaers et al., 2009; Sanders et al., 2025; Tuomisto et al., 2012), yet the scope has been restricted to nitrogen (N), phosphorus (P), pesticides, soil erosion, and/or geographically limited.

The production of food using organic or conventional agriculture differs in both legal and cultural aspects that vary by region. The major differences lie in the types of fertilizer applied and the techniques for managing pests and weeds. Modern organic agriculture, sometimes called biological or ecological agriculture, eliminates most synthetic fertilizers, pesticides, and herbicides used in conventional agriculture (Reganold & Wachter, 2016). According to the US National Organic Program, most synthetic compounds including nutrients, pesticides, or herbicides cannot be applied to certified organic fields (7 eC.F.R. §205.105 2025 [where eC.F.R. stands for electronic Code of Federal Regulations]). There are several exceptions to prohibited synthetic substances provided these substances “do not contribute to contamination of crops, soil, or water.” There are also prohibited non‐synthetic substances. Most nonagricultural substances and nonorganic agricultural substances such as sewage sludge (biosolids) are also prohibited. Thus, because the synthetic fertilizers typically used in conventional agriculture such as urea ammonium nitrate are prohibited, to meet crop nutrient demands, organic growers rely on carbon‐based fertilizers including compost, manures, and legume plants (Fabaceae), which foster biological nitrogen fixation. In place of pesticides and herbicides to reduce pests and weeds, organic agriculture growers often use natural pest and weed management such as soil tillage or roller crimpers, and increase crop diversity through adjacent or rotational plantings (Reganold & Wachter, 2016).

Studies of soil health indicate that practices associated with organic agriculture benefit the physical, chemical, and biological properties of soil, enhancing soil health (Christel et al., 2021; Tully & McAskill, 2020; Wijesinghe et al., 2023). In comparison with conventionally farmed soils, organic agriculture has been shown to yield greater soil organic carbon, improve soil quality, and reduce soil erosion (Reganold & Wachter, 2016). Soil health benefits are often assumed to confer water quality benefits. This premise is reasonable, yet a holistic understanding of the net effect on water quality is not well established. For several pollutants, well‐established mechanisms suggest that organic agriculture can influence water quality differently than conventional systems (Cambardella et al., 2015; Seufert & Ramankutty, 2017; Sivaranjani & Rakshit, 2019). For example, transitioning to organic production is expected to reduce the presence of most synthetic pesticides and herbicides in water leaving agricultural fields, as these substances are generally prohibited under organic certification standards and therefore not applied by growers (Lemke et al., 2024; Reganold & Wachter, 2016; Seufert & Ramankutty, 2017).

Mechanisms for nitrogen, phosphorus, and sediment pollution also have potential to differ in conventional and organically managed systems depending on the type of fertilizer applied and tillage and cover crop approaches. Inorganic nitrogen forms (nitrate, nitrite, and ammonium) are highly soluble and readily mobilized in soil water. As a result, systems that rely on synthetic inorganic nitrogen (conventional systems) or fresh manure (used in both conventional and organic systems) can easily lose these forms of nitrogen through runoff and leaching (Cameron et al., 2013; Ghaly & Ramakrishnan, 2015). Leachate is the water solution that contains some soil components as dissolved or suspended constituents, which are collected during the downward movement of water through the soil profile, and is commonly measured using lysimeters installed at various depths. To mitigate these losses, organic systems more frequently employ “catch crops,” sometimes 3.2–12.1 times more often than conventional systems to retain inorganic nitrogen (Barbieri et al., 2017; Sieling, 2019). However, if the timing of catch crop decomposition is not synchronized with subsequent crop uptake, these systems can shift from nitrogen retention to nitrogen release, thereby contributing to pollution. Additionally, evidence from recently transitioned fields suggests that shifts from conventional to organic management can reduce nitrification rates and, consequently, nitrate export (Price et al., 2025). These responses, however, tend to emerge gradually and are strongly influenced by interannual weather variability, which should be explicitly accounted for in nutrient dynamics and modeling efforts for agriculture practices (Price et al., 2025).

Phosphorus lost from agricultural fields is often particle‐bound (60%–90% [Sharpley et al., 1995]) and, thus, tightly connected with erosion‐related losses. Fertilizer type further complicates phosphorus management: for instance, manure sources such as chicken manure often contain high phosphorus relative to nitrogen, making it difficult to achieve balanced N:P:K ratios in organic systems without careful selection and combination of inputs (McDowell, 2012; Nelson & Janke, 2007). Over time, repeated applications can lead to phosphorus accumulation in soils, increasing the risk of loss as the soil's sorption capacity becomes saturated (McDowell, 2012).

**Core Ideas**

- There are more cases of lower nitrogen or soil aqueous export under organic agriculture.
- There are more cases of reduced phosphorus or carbon aqueous export under conventional agriculture.
- Uncertainty remains in the water quality effects from different agricultural managements.

More broadly, management practices that influence soil organic matter, nutrient pools, and aggregate stability also shape nutrient retention and loss pathways. Organic systems often promote higher soil organic matter, improved aggregation, and continuous ground cover, all of which can reduce erosion and associated phosphorus losses (Ghabbour et al., 2017; Seitz et al., 2019). Nevertheless, tillage (used in both organic and conventional systems) can counteract these benefits by increasing soil disturbance and erosion risk. Therefore, practices that minimize tillage or maintain consistent cover, regardless of farming system, are particularly effective in reducing sediment and nutrient losses. Given the uncertainties at which these combined mechanisms scale to in situ situations that vary in soil types and latitudes, their overall impact on water quality, including nutrients and sediment, remains unclear.

Nonetheless, the downstream effects of these water quality pollutants are considerable. Nitrate from leaching has the potential to contaminate drinking water intake sources, and nitrate is regulated in drinking water with the NO_3_‐N maximum concentration in the United States at 10 mg NO_3_‐N L^−1^ (USEPA, n.d.) and the World Health Organization maximum for drinking water at 11.3 mg NO_3_‐N L^−1^ (World Health Organization, 2022). In addition to human health concerns regarding drinking water, nitrogen and phosphorus losses can contribute to eutrophication, which is a major issue in Chesapeake Bay and Baltic Sea among other coastal ecosystems (Carpenter et al., 1998; Malone & Newton, 2020; Pan et al., 2021). Increased sedimentation can bury important fish reproductive habitats and deplete macrophytes and macroinvertebrates, while some pesticides are also documented to reduce metrics of fish reproduction and increase mortality of freshwater macroinvertebrates (Cooper, 1993).

In this review, we synthesize findings from comparative studies of terrestrial row‐crop organic and conventional agricultural systems, focusing on surface runoff, shallow subsurface runoff, and soil leachate water quality. This review aims to identify the consequences that conventional and organic agricultural systems have on surface water quality with the goal of expanding our understanding about agricultural system‐specific impacts beyond the focus of soil health and beyond the scope of specific pollutants or regions. While the literature search did not target specific pollutants, data for nitrogen, phosphorus, and sediment were most commonly reported and, thus, a focus of the trends reported in this review. In addition to the water quality pollutants, the runoff water volume was also commonly reported and is included in the scope of this analysis.

## 2 MATERIALS AND METHODS

### 2.1 Water quality literature methods

Suitable papers for inclusion in this review met the following criteria: (1) the study had a paired organic (per definition of organic as deemed by the country in which the study occurred) and conventional treatment (meta‐analyses and landscape scale studies of all farms in a region were excluded; modeling and simulations were included; replication was not a requirement), (2) the study was conducted using row‐crops (e.g., organic dairy farms and flooded rice [*Oryza sativa* L.] paddy trials were excluded), and (3) surface water quality parameters were measured (groundwater wells and strictly hydrologic studies such as infiltration were out of scope for this review).

Using Web of Science, we searched all databases on July 11, 2019, and reran our search on September 6, 2023, May 10, 2024, and July 9, 2025, to cover the time periods that had elapsed using the following search parameters and excluded retracted papers and the preprint citation index: Topic (water AND agriculture AND organic AND farming). We did not restrict the search to a timespan or by location. These searches yielded 9617 results, which were then refined using the terms “physical” (1638 results), “chemical” (3362 results), or “biological” (1599 results) for a total of 4853 results. The titles of these results were checked for relevancy, and 437 titles were retained. The abstracts were then sorted, and 132 of these appeared to be relevant. Of these 132 papers, we could access all but four, and after examination, 24 were determined suitable. Twenty‐four additional papers were collected and evaluated for suitability because they were cited within our initial set of accepted papers and from review papers, and 13 of these were determined suitable for inclusion. In total, 37 papers met the criteria and were included in this review.

### 2.2 Data collection

We then collected information about the treatments in the studies. For the background information, we marked study location, soil type, length of time the treatment had been organically or conventionally farmed, the years of data collection, the crops and rotations, methods of weed and other pest control, irrigation, field slope, and average annual precipitation. From the study treatments, we recorded the type and amounts of fertilizer, other inputs, non‐crop plants including green manure and cover crops, pesticide type and application frequency, tillage, and number of replicate fields. We additionally recorded values of soil nitrogen, soil phosphorus, and soil organic matter. We noted the methods used for data collection and marked all response variables in the studies that related to water quality.

To evaluate water quality influences from the organic and conventional managements, we recorded the water quality results and conclusions from each study, combining literature information if they were published from the same field trials. If data were in graphical form, we used Graphreader (version 1) to extract the data, and for one paper that presented data with a boxplot, we confirmed data normality and calculated standard error using standard deviation as the interquartile range divided by 1.35. To consolidate data for comparisons across studies, we conducted calculations to obtain similar units where possible. Calculations included converting concentration to load (mass per area per time as kg ha^−1^ per year, hour, or minute depending on the experimental information available), converting to the same molecular mass (e.g., NO_3_‐N instead of NO_3_), or converting to the same scale in the unit (e.g., kg instead of g). To determine the percentage change between organic and conventional treatments, we subtracted the difference in values and divided by the conventional value using the following equation:

$$\begin{array}{c} \%\;\mathrm{Change}\;\mathrm{from}\;\mathrm{conventional}\;\mathrm{value}\;=\frac{\mathrm{Average}\;\mathrm{organic}\;\mathrm{value}-\mathrm{Average}\;\mathrm{conventional}\;\mathrm{value}}{\mathrm{Average}\;\mathrm{conventional}\;\mathrm{value}}\;\;\times \;100\% \end{array}$$

## 3 RESULTS

### 3.1 Scope of included papers

The papers used from our water quality literature search came from studies primarily conducted in Europe (28 papers, 76%) and North America (nine papers, 24%) (Tables S1 and S2). Roughly half of the studies specified that the main soil types were silty loam and sandy loam. Seventeen studies reported the field slope, which ranged between 0% and 20%, with an average of 5%. Annual average precipitation ranged from 132 to 1105 mm, with an average of 740 mm reported across the 23 studies that included this information. Only seven studies (19%) reported using irrigation. A variety of grain crops including corn (*Zea mays* L.), wheat (*Triticum aestivum* L.), and soybean (*Glycine max* (L.) Merr. were used in 81% of studies, but 49% of studies had additional or exclusively non‐grain crops (primarily potatoes [*Solanum tuberosum* L.]). Organic weed control typically used a mechanical form of tillage (roto tilling, hoeing, disking reported in 54% of studies), though some studies used hand weeding, plastic film, or organic‐approved pesticides (reported in 16% of studies). Most nonorganic treatments used herbicides for weed control (59% reported), and some combined this with mechanical tilling, hand weeding, or plastic film (16% reported). The final literature set included several subsurface drainage pipe systems used to collect subsurface water. These studies were Literature IDs 130/E37/E38, E22, E24, 56, 202, and E10. Additional study characteristics are included in Table S2.

### 3.2 Impacts of organic agriculture on water quality

Our literature synthesis of paired field studies revealed that agricultural practices affected multiple dimensions of water quality, ranging from macronutrient losses through leachate to the volume and turbidity of runoff. The direction and magnitude of these changes were wide‐ranging, and the surveyed body of research encompassed considerable variations in the specific agricultural management techniques, duration of the agricultural practice, and soil types. Nitrogen loss to surface and groundwater was the focus of most studies evaluating water quality from agricultural practices (27 studies), and nitrate was particularly emphasized (Table 1). Phosphorus loss was the second most commonly studied aspect (12 of the 37 total papers). Most parameters were reported in only one study: of the 110 total combinations of variables and units measured, 70 of the conventional and 62 of the organic field variable–unit combinations (e.g., NO_3_‐N mg L^−1^ in runoff) were only reported in a single study (*n* = 1) (Table S2).

**TABLE 1:** Percent change of organic agriculture value relative to paired conventional agriculture value.

		Change from conventional treatment				
Variable	*n*	Average (%)	Median (%)	S.D.	S.E.	C.I.
Nitrogen						
N, leached	28	−4.06	−5.71	29.8	5.63	−15.6 to 7.48
N, leaching potential	4	2.97	−4.22	48.1	24.1	−73.6 to 79.5
NO_3_‐N, leached	26	−34.8	−43.72	39.2	7.69	−50.6 to −19.0
NO_3_‐N, in soil water	1	25.0	–	NA	NA	NA
NO_3_‐N + NH_4_‐N, leached	4	−26.2	−27.5	19.1	9.54	−56.6 to 4.10
NH_4_‐N, drainage	2	16.7	–	23.6	16.7	−195 to 228
NH_3_, drainage	1	−46.2	–	NA	NA	NA
TN, leached	6	2.32	−3.95	37.6	15.3	−37.1 to 41.7
TKN, leached	3	32.4	22.0	18.0	10.4	−12.3 to 77.1
N, runoff	3	16.70	29.2	21.6	12.5	−37.0 to 70.4
TN, runoff	2	−0.83	–	25.1	17.7	−226 to 225
NO_3_‐N, runoff	8	−28.5	−45.7	62.3	22.0	−80.6 to 23.6
TKN unfiltered, runoff	8	−25.6	−32.6	54.5	19.3	−71.1 to 19.9
Dissolved TKN, runoff	4	−0.90	20.1	51.1	25.5	−82.2 to 80.4
TDN, runoff	1	−56.7	–	NA	NA	NA
DON, runoff	4	3.18	25.6	52.6	26.3	−80.6 to 86.9
NH_4_‐N, runoff	3	17.3	34.2	62.9	36.3	−139 to 174
NH_3_, runoff	1	53.1	–	NA	NA	NA
Phosphorus						
TP, leached	10	32.2	25.0	64.0	20.2	−13.6 to 78.0
PO_4_^3−^, leached	4	−2.4	16.7	51.6	25.8	−84.5 to 79.8
TP, runoff	10	17.9	−27.6	149	47.1	−88.6 to 124
Dissolved reactive P, runoff	5	23.3	26.3	64.4	28.8	−56.8 to 103
Dissolved TP, runoff	5	78.8	39.8	112	49.9	−59.9 to 217
DOP, runoff	4	104	92.2	134	66.9	−109 to 317
PO_4_^3−^, runoff	2	36.4	–	27.9	19.7	−214 to 287
P erosion	1	−56.7	–	NA	NA	NA
P sediment	1	−50.0	–	NA	NA	NA
Carbon						
C, runoff	8	96.8	48.9	215	76.2	−83.3 to 277
TOC, runoff	4	31.2	45.9	65.7	32.8	−73.3 to 136
C erosion	1	−54.1	–	NA	NA	NA
HCO_3_^−^, leached	2	25.5	–	27.1	19.2	−218 to 269
Runoff						
Runoff	23	−1.28	−6.49	66.3	13.8	−29.9 to 27.4
Runoff, coefficient	2	−100	–	0	0	0–0
Runoff, predicted	1	10.2	–	NA	NA	NA
Soil loss						
Soil loss, runoff	9	−1.8	−62.8	189	63	−147 to 144
TSS, runoff	6	−10.9	−68.3	145	59.0	−163 to 141
Water erosion	2	−68.1	–	8.9	6.3	−148 to 12.1
Sediment delivery	4	190	79.1	350	175	−367 to 747
Sediment detachability	2	18.0	–	3.77	2.67	−15.9 to 51.9
Soil loss, drainage	2	−17.1	–	12.7	8.99	−131 to 97.1
Ecotoxicity						
Aquatic ecotoxicity ha^−1^	4	−81.8	−81.9	2.64	1.32	−86.0 to −77.6
Pesticide, runoff	2	−68.6	–	16.2	11.4	−213 to 76.6
2,4‐D, runoff	2	−1.84	–	0.08	0.06	−2.60 to −1.08
Bromoxynil, runoff	2	11.1	–	4.21	2.98	−26.7 to 48.9
Chlorothalonil^a^_,_ soil water	1	−100	–	NA	NA	NA
Clopyralid, runoff	2	−96.1	–	2.58	1.83	−119 to −72.9
Cu, leached	1	0	–	NA	NA	NA
DDD^b^, soil water	1	−100	–	NA	NA	NA
DDE^c^, soil water	1	−94.7	–	NA	NA	NA
Dicamba, runoff	2	50.4	–	156	110	−1351 to 1453
Flusilazole^d^, soil water	1	−100	–	NA	NA	NA
HCH^e^, soil water	1	−100	–	NA	NA	NA
MCPA, runoff	2	−17.5	–	7.92	5.60	−88.7 to 53.6
Pendimethaline^f^, soil water	1	−81.8	–	NA	NA	NA
Triallate, runoff	2	−44.0	–	0.06	0.04	−44.5 to −43.5
Trifluralin, runoff	2	−73.6	–	4.17	2.95	−111 to −36.2
Trifluralin^g^, soil water	1	−100	–	NA	NA	NA
Zn, leached	1	22.2	–	NA	NA	NA
Miscellaneous						
B, leached	1	22.2	–	NA	NA	NA
Ca^2+^, leached	2	18.5	–	26.5	18.7	−219 to 256
Cl^−^, leached	2	2.82	–	22.5	15.9	−200 to 205
TK, leached	4	−43.6	−44.3	10.7	5.34	−60.6 to −26.6
K, leached	2	−2.15	–	32.0	22.7	−290 to 286
K, runoff	2	16.8	–	21.2	15.0	−174 to 208
K^+^, leached	2	−2.54	–	20.0	14.1	−182 to 177
Mg^2+^, leached	2	42.5	–	41.5	29.4	−331 to 416
Mn, leached	1	0	–	NA	NA	NA
SO_4_^2−^, leached	2	24.6	6.85	25.2	17.8	−201 to 251
Aquatic eutrophication potential^h^	8	−24.6	−19.9	32.7	11.5	−51.9 to 2.68

*Note*: Negative numbers indicate lower values were measured in the organic treatment. *n* is the number of paired conventional and organic treatment comparisons, not the number of studies. – indicates no difference from the mean value.

Abbreviations: 2,4‐D, 2,4‐dichlorophenoxyacetic acid; C.I., confidence interval at 95%, lower bound to upper bound; DDD, dichlorodiphenyldichloroethane; DDE, dichlorodiphenyldichloroethylene; DON, dissolved organic nitrogen; DOP, dissolved organic phosphorus; HCH, hexachlorocyclohexane; MCPA, 4‐chloro‐2‐methylphenoxy acetic acid; NA, not applicable; S.D., standard deviation; S.E., standard error from the mean; TDN, total dissolved nitrogen; TK, total potassium; TKN, total Kjeldahl nitrogen; TN, total nitrogen; TOC, total organic carbon; TP, total phosphorus; TSS, total suspended solids.

^a^ Fungicide organochlorine.

^b^ Metabolite organochlorine.

^c^ Metabolite organochlorine.

^d^ Pesticide triazole.

^e^ Insecticide organochlorine.

^f^ Herbicide organonitrate.

^g^ Herbicide organochlorine.

^h^ Risk for a water body becoming overly enriched with nitrogen or phosphorus.

### 3.3 Water chemistry: Nitrogen

**FIGURE 1:**
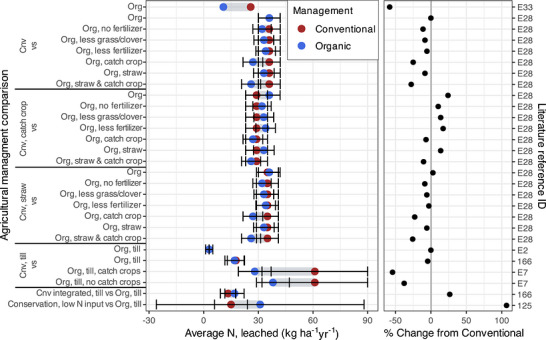
Average leached nitrogen (N) from conventional and organic managements from each study and percent that organic management average leached nitrogen changed relative to the conventional average leached nitrogen recorded in the study. Agricultural management comparisons: Cnv is conventional agriculture and Org is organic agriculture. Error bars are standard error from the mean.

Of the 28 paired conventional and organic comparisons for nitrogen leaching, 64% of comparisons showed a reduction in nitrogen leached from organic fields and 7% had no change (Figure 1). The average reduction in nitrogen leached from organic fields was 4% (S.E. [standard error from the mean] = 5%, median = −5.71%, Table 1). All of the leached nitrogen values were given or able to be calculated as mass per unit area per year. The average leached nitrogen for organic and conventional fields was 26.2 kg N ha^−1^ year^−1^ from conventional fields (*n* = 9, median = 25.8 kg N ha^−1^ year^−1^) and 26.8 kg N ha^−1^ year^−1^ from organic fields (*n* = 13, median = 31.0 kg N ha^−1^ year^−1^) (Table S3). Both treatments had the same minimum leached nitrogen values (3 kg N ha^−1^ year^−1^), but the maximum value for conventional treatments exceeded that of organic treatments by 1.5 times (61 kg N ha^−1^ year^−1 ^vs. 38 kg N ha^−1^ year^−1^). Soil nitrogen was greater in the organic system for 17 of 24 comparisons, but this did not indicate which treatment would have greater nitrogen loss: seven of those comparisons had more nitrogen leached from organic fields, and 10 comparisons had more nitrogen leached from conventional fields.

For runoff, three paired comparisons were available for nitrogen, two for total nitrogen (TN), eight for unfiltered total Kjeldahl nitrogen (TKN), and one for total dissolved nitrogen. From those, almost two‐thirds of the comparisons had reduced loss with the organic treatment. The mean change from conventional treatments averaged from 17% to −79%. The average nitrogen lost from either agricultural treatment through runoff was considerably lower than that lost through leaching. Conventional treatments lost an average of 4.4 kg N ha^−1^ year^−1^ to runoff, while organic treatments lost an average of 5.8 kg N ha^−1^ year^−1^ (Table S3). The exception was one value of unfiltered runoff TKN lost from conventional fields at 19 kg N ha^−1^ year^−1^.

**FIGURE 2:**
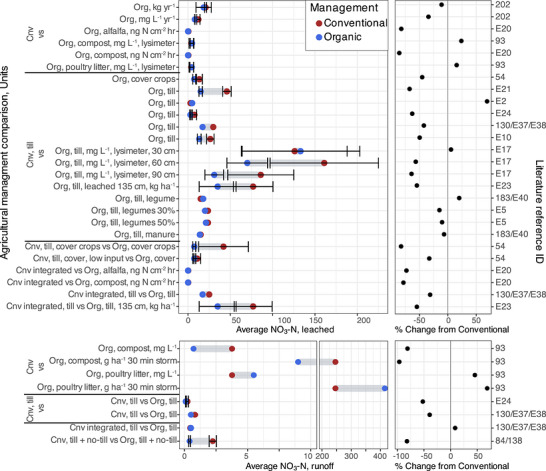
Average nitrate (NO_3_‐N) lost as leachate or runoff from conventional and organic managements from each study, and percent that organic management average nitrate loss changed relative to the conventional average nitrate loss as recorded in the study. Units are kg N ha^−1^ year^−1^ unless otherwise stated. Agricultural management comparisons: Cnv is conventional agriculture and Org is organic agriculture. Error bars are standard error from the mean.

Nitrate losses through leaching or runoff decreased in the organic agricultural field treatment for most of the paired comparisons, and leaching was the primary mode of nitrate loss (Figure 2). Nitrate leaching from the organic paired field was lower than from the conventional field in 81% of paired study comparisons (21 of 26 comparisons). On average, nitrate leached from organic treatments was roughly one‐third less than that from the paired conventional treatment (median = −44%, Table 1). Just under half of the nitrate loss measurements were calculated as a mass loss per unit area per unit time. For these units, organic fields leached an average of 13.4 kg NO_3_‐N ha^−1^ year^−1^ (*n* = 10), while conventional fields leached 22.1 kg NO_3_‐N ha^−1^ year^−1^ (*n* = 11) (Table S3). Four measurements were made of nitrate concentrations collected from lysimeters installed at different depths, and one study collected leachate during a rainfall simulation. Twenty‐two comparisons included soil nitrogen as TN or nitrate, and there was not an association between soil nitrogen and the direction of change for organic fields relative to conventional for average leached nitrate. Three of the five comparisons with worse nitrate leaching from organic treatments had higher soil nitrogen in the organic fields, while two of 19 comparisons with worse nitrate leaching from the conventional treatment had higher soil nitrogen.

Nitrate in runoff was also lower from organic fields in five of the eight paired organic–conventional comparisons (Figure 2). Similar to nitrate in leachate, nitrate in runoff decreased by roughly one‐third from conventional to organic fields (median = −46%, Table 1). Nitrate in runoff was also predominantly available as mass lost per area over a year, although concentration and rainfall simulation measurements were also reported. Considering the mass loss measurement, conventional fields lost an average of 0.946 kg NO_3_‐N ha^−1^ year^−1^ (*n* = 4, median = 0.655 kg NO_3_‐N ha^−1^ year^−1^) through runoff, while organic fields lost 0.342 kg NO_3_‐N ha^−1^ year^−1^ (*n* = 3, median = 0.405 kg NO_3_‐N ha^−1^ year^−1^) (Table S3). Seven of the eight studies had no difference in soil nitrogen.

In contrast to patterns observed for nitrate, ammonium in drainage and runoff from organic fields increased by roughly 17% (runoff median = 34%, Table 1). Although ammonium may be elevated in fertilizer additions, it comprised only 4% of the conventional system and 6% of the organic system nitrogen lost as a mass per hectare per year. Data availability for ammonium was limited, resulting in just five paired comparisons for leachate and runoff combined. On average, ammonium leached from organic fields at amounts similar to conventional fields (0.08 kg NH_4_‐N ha^−1^ year^−1^ [*n* = 2] vs. 0.07 kg NH_4_‐N ha^−1^ year^−1^ [*n* = 1]). Ammonium in runoff from conventional fields was greater on average than that from organic fields (0.92 kg NH_4_‐N ha^−1^ year^−1^ [*n* = 3] vs. 0.75 kg NH_4_‐N ha^−1^ year^−1^ [*n* = 2]). Ammonia was even further limited in data availability, with one paired comparison each for drainage and runoff that yielded opposite trends. For drainage, ammonia decreased by roughly half and for runoff, ammonia increased by roughly half from the conventional to the organic field.

### 3.4 Water chemistry: Phosphorus

**FIGURE 3:**
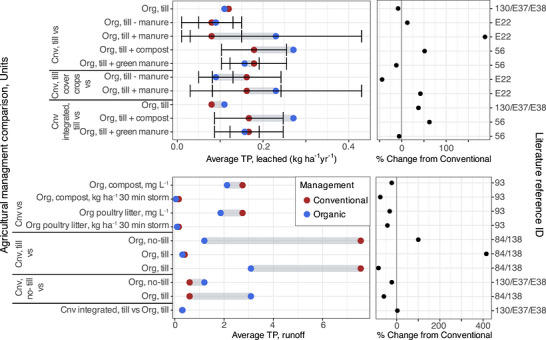
Average total phosphorus (TP) lost as leachate or runoff from conventional and organic managements from each study, and percent that organic management average TP loss changed relative to the conventional average TP loss as recorded in the study. Agricultural management comparisons: Cnv is conventional agriculture and Org is organic agriculture, and units are kg ha^−1^ year^−1^ unless otherwise stated. Error bars are standard error from the mean.

Relative to the paired conventional field, organically farmed fields typically had lower total phosphorus (TP) losses through runoff (70% of paired comparisons) but higher losses through leaching (60% of paired comparisons) (Figure 3). For TP in runoff, one paired comparison increased 415% from conventional to organic treatments which outweighed the moderate decreases in TP from the other comparisons. Thus, overall, the average TP lost in runoff was 18% greater from organic treatments, but the standard error was high (47%), and the median TP lost indicated that organic treatments losses were lower than the conventional treatments by 27% (Table 1). Leached TP was on average 32% greater in organic treatments (median = 25%, Table 1). Phosphate in both runoff and leachate was highly variable. Leached phosphate increased with the organic treatment in two of four paired comparisons by 33%, while the other two showed no difference or decreased by 76%. Phosphate loss from runoff increased from organic fields in all two paired comparisons by 36% on average. Soil phosphorus concentrations did not inform which agricultural system would have more leached TP. However, for TP in runoff, six of seven comparisons with improved losses from organic systems tended toward higher soil phosphorus in the organic system.

In general, more TP and phosphate were lost through runoff than through leaching (Table S3). Runoff phosphate was an average of 0.32 kg PO_4_^3−^ ha^−1^ year^−1^ (*n* = 2) from conventional treatments and 0.43 kg P ha^−1^ year^−1^ (*n* = 1) from organic treatments. Leached phosphate was an average of 0.13 kg PO_4_^3−^ ha^−1^ year^−1^ (*n* = 4) from conventional treatments and 0.12 kg PO_4_^3−^ ha^−1^ year^−1^ (*n* = 3) from organic treatments. Mean TP lost from conventional fields in runoff was 2.21 kg P ha^−1^ year^−1^ (*n* = 4) compared with 1.53 kg P ha^−1^ year^−1^ (*n* = 3) in organic fields, but the medians showed an opposite trend with 0.50 kg P ha^−1^ year^−1^ lost from conventional fields and 1.2 kg P ha^−1^ year^−1^ lost from organic fields. Mean TP lost from conventional fields from leaching was 0.13 kg PO_4_^3−^ ha^−1^ year^−1^ (*n* = 6) compared with 0.17 kg P ha^−1^ year^−1^ (*n* = 5) in organic fields.

### 3.5 Water chemistry: Carbon

Carbon (C) in runoff increased from organic treatments by 97% on average (S.E. = 76%, *n* = 8, median = 49%, Table 1). However, the mean value of carbon in runoff for studies that measured it per unit area was nearly double in conventional treatments 41 kg C ha^−1^ year^−1^ (*n* = 2) compared with organic treatments (21 kg C ha^−1^ year^−1^ [*n* = 2, Table S3]). Total organic carbon in runoff also increased in 75% of organic treatments, but only by 31% on average (*n* = 4, median = 46%, Table 1). Despite this, only one comparison had a difference in soil carbon and reported higher values for the organic treatment.

### 3.6 Water chemistry: Pesticides

Organic treatments generally reduced the detection of pesticides in samples or the concentrations of those pesticides (Table 1 and Table S3). In organic treatments, overall aquatic toxicity potential from 1,4‐dichlorobenzene decreased by 81% (*n* = 4). All of the other pesticides (2,4‐D [2,4‐dichlorophenoxyacetic acid], clopyralid, MCPA [4‐chloro‐2‐methylphenoxy acetic acid], chlorothalonil, dichlorodiphenyldichloroethane, dichlorodiphenyldichloroethylene, flusilazole, hexachlorocyclohexane, pendimethaline, trifluralin, and triallate) also decreased in the organic treatments except for dicamba and bromoxynil, which were detected in 50% and 11% more water samples in organic treatments, respectively (Table 1).

### 3.7 Water physical properties: Soil erosion by water

**FIGURE 4:**
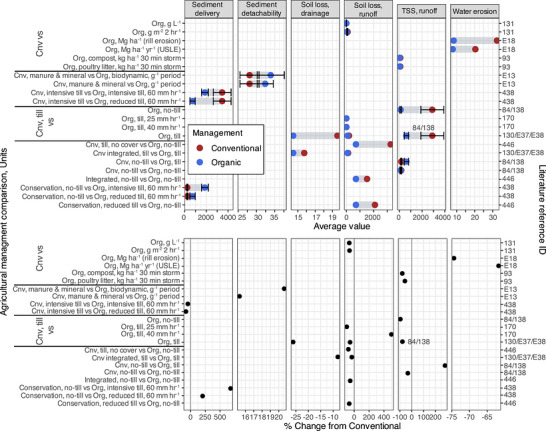
Average sediment or soil lost from conventionally and organically managed fields from each study, and percent that organic management average sediment or soil loss changed relative to the conventional average sediment or soil loss as recorded in the study. Agricultural management comparisons: Cnv is conventional agriculture and Org is organic agriculture. Units are kg ha^−1^ h^−1^ unless otherwise stated. Labels on y‐axis with mm h^−1^ refer to rainfall intensity. Error bars are standard error from the mean. Literature reference ID listed on graph for one total suspended solids (TSS), runoff pair.

Soil loss was most frequently greater from conventional fields than organic fields as measured through sediment delivery and detachability, soil loss in drainage and runoff, total suspended solids (TSS), and water erosion models (Figure 4). Soil loss through runoff decreased in organic treatments in 89% of paired comparisons. The average soil loss difference between treatments was minimal at −1.8% with a wide range of values (S.E. = 63%, median = 63%) (Table 1), driven particularly by one study (Literature Reference ID 170, Table S1). For studies calculating annual budgets, soil loss was an average of 1437 kg ha^−1^ year^−1^ (*n* = 5) from conventional fields and 393 kg ha^−1^ year^−1^ (*n* = 2) from organic fields (Table S3). TSS in runoff decreased in organic treatments in 83% of paired comparisons (Figure 4). The mean TSS in runoff was 1532 kg ha^−1^ year^−1^ in conventional treatments and 363 kg ha^−1^ year^−1^ in organic treatments as averaged from two comparisons (Table S3). Sediment delivery measurements taken all from one rainfall simulation study showed mixed results based on tillage, with two comparisons indicating greater sediment delivery from conventional intensive till treatments and two comparisons indicating greater sediment delivery from organic treatments compared with conventional (conservation no‐till) treatments (Figure 4). In all but two of the 21 comparisons for soil erosion by water that reported soil organic matter or soil carbon content, values were higher in the organic systems.

When soil tillage was accounted for, patterns of soil loss depended on tillage practices. When both organic and conventional fields were tilled, eight of the nine pair‐wise comparisons showed less soil was lost from the organic treatment (*n* = 9). For the two studies where both treatments were no‐till, soil losses still decreased in the organic treatment. In one of these studies, this result could be attributed to soil carbon as TSS loss was negatively correlated with soil carbon (Larsen et al., 2014). In the other study, the result could be due to differences in plant cover extent: the organic treatment had vegetation or legume cover for the entire soil surface, while the integrated no‐till conventional treatment used 3‐m‐wide strips (Zuazo et al., 2020). When comparing across tillage intensities, the no‐till practices reduced soil loss regardless of the use of conventional or organic agriculture (*n* = 3 each for organic no‐till vs. conventional till and conventional no‐till vs. organic till).

### 3.8 Water physical properties: Runoff volume

**FIGURE 5:**
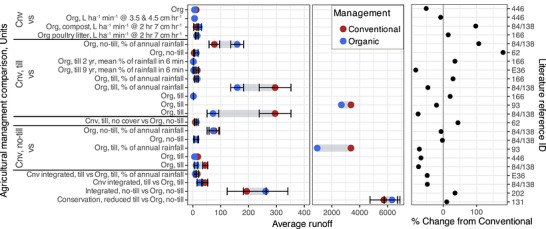
Average water runoff from conventionally and organically managed fields from each study, and percent that organic management average runoff changed relative to the conventional average runoff as recorded in the study. Agricultural management comparisons: Cnv is conventional agriculture and Org is organic agriculture, and units are mm year^−1^ unless otherwise stated. Labels on y‐axis with cm h^−1^ refer to rainfall intensity. Error bars are standard error from the mean

Runoff from the organic treatments decreased compared to the conventional treatments for just over half of the paired comparisons (57%), while for 43% of comparisons, runoff was greater in the organic treatment (Figure 5). The percent change in runoff between paired comparisons decreased only slightly for organic treatments (−1.28%, *n* = 23, Table 1). Runoff was reported using a variety of approaches including percent of annual rainfall and from rainfall simulation studies, but the most commonly used unit was volume per year (*n* = 10 in conventional and *n* = 6 in organic). Using the volume per year calculation, average runoff volumes from all organic treatments decreased relative to all conventional treatments (64 mm year^−1^ vs. 88 mm year^−1^) (medians = 13 mm year^−1^ vs. 44 mm year^−1^, respectively) (Table S3). Runoff as a percentage of annual rainfall was 15% (*n* = 4) from conventional fields and 12% (*n* = 3) from organic fields (medians = 7% and 11%, respectively).

## 4 DISCUSSION

This review indicates that for most paired studies of terrestrial row‐crops managed under organic or conventional practices, nitrogen and soil losses from leaching and runoff improved under organic management, while phosphorus and carbon exports were less with conventional management. Yet, even for these variables, one system of agriculture was not exclusively an improvement over the other. Rather, the influence of conventional and organic agriculture on water quality depended on the specific practices used in each system, such as the type of fertilizer and use of tillage, and was not clearly dictated by differences in soil nutrient content. For many water quality variables, though, comparisons were not robust due to the scarcity of data that met our criteria. As robust comparisons arise from future research, an important consideration will be how water quality consequences scale with differences in the land area required to provide equivalent yields as this will be critical to understanding the full environmental footprint of different agricultural systems.

Nitrate was the primary variable where the loss in leaching and runoff from agricultural fields was reduced with organic farming systems. For leached nitrate, this pattern relied on 26 paired‐comparisons, making it one of the most strongly supported, whereas the comparisons for nitrate in runoff were less than 10. This pattern aligns with the meta‐analysis of only European studies by Tuomisto et al. (2012) that also identified lower nitrogen leaching from organic fields per unit of area and with a systematic review by Sanders et al. (2025) that found lower nitrate leaching in 45% of comparisons (and higher nitrate leaching in 19% of comparisons) from 386 comparisons. While our review found leached nitrate was reduced by an average of one‐third with organic agriculture, the average mass loss from organic fields was still 13 kg NO_3_‐N ha^−1^ year^−1^. The load reductions necessary to improve water quality can vary considerably, and a catchment‐level lens is needed to understand whether reductions that stem from shifting agricultural systems will have a major influence. This context is important to consider as some policies push for a shift from conventional to organic systems in order to reduce nitrate inputs. For example, part of the action plan to decrease nitrate leaching includes incentivizing a transition to organic agriculture (e.g., Denmark government [The Danish Government, 2021]). And the seven jurisdictions around Chesapeake Bay have enacted targets to meet USEPA's Chesapeake Bay Total Maximum Daily Loads, which would decrease TN by 25% relative to 2009 levels to 185.9 million pounds of nitrogen. These policy efforts highlight the importance of quantifying impacts from changes in the agricultural sector.

At first glance, the justification for policies that seek to improve water quality by shifting agricultural systems aligns with our findings that organic systems could reduce nitrate leaching. Yet, our results showed that in a fifth of comparisons, the organic system was *not* lower than the conventional system in leaching nitrate, indicating that the specific practices used or characteristics of the system are an important consideration. The reason for the reduction in nitrate losses with organic agriculture may lie in differences of fertilizer and crop types, which may or may not be unique to a type of agricultural system. Originally, organic agriculture often used carbon‐based fertilizers such as compost or green manure that are slower to release nitrogen through decomposition (Seufert et al., 2017). This slow‐release allows for more complete uptake by plants and microorganisms, provided there is synchrony in crop demand and nutrient mineralization (Singh et al., 2001). This leaves nitrate, which is highly soluble in water, less susceptible to loss through water pathways like leaching and runoff, and could be the reason for the reduction in nitrate losses. Conventional agriculture frequently uses inorganic fertilizers that include ammonium and nitrate and, therefore, have a higher potential for the nitrogen to dissolve in water and be exported from fields with runoff or leaching. However, there are some carbon‐based fertilizers used in both organic and conventional agriculture, such as fresh manure slurry, and especially poultry manure, that contain highly soluble inorganic nitrogen. We found that accounting for these types of fertilizer can influence the outcome of the paired comparisons. For example, relative to conventional management in the same study, organic management had 81% lower nitrate loss in runoff when organic compost was used, but 45% higher nitrate loss in runoff when organic poultry litter was used (Evanylo et al., 2008). From 3 years of nitrogen analysis in the two sources of fertilizer, this study found that poultry litter contained 7.7–13.1 NH_4_‐N (g kg^−1^) and 0.0–0.3 NO_3_‐N (g kg^−1^), whereas the compost contained 0.1 to undetected NH_4_‐N (g kg^−1^) and 1.1–0.1 NO_3_‐N (g kg^−1^). Many considerations beyond fertilizer that may or may not be unique to an agricultural system are also important factors in controlling nitrate leaching. Simmelsgaard (1998) identified drainage, soil type, nitrogen input level, and the type, timing, and duration of crops or cover in the field as major factors determining nitrate loss and concluded that once nitrate has leached past the rooting zone, it is typically lost from the soil system. These important distinctions could enhance our ability to reduce nitrogen pollution by increasing the specificity of recommendations beyond a generalized agricultural system.

Phosphorus is another macronutrient of concern for water quality, and we found that the primary routes of TP loss through leaching or runoff may differ by agricultural system. Designing these studies to incorporate a full mass balance from multiple loss pathways would tease apart the drivers of loss in agricultural systems and allow for targeting surface and groundwater concerns separately. However, we found few studies that measured these multiple loss pathways (an exception was a study published as reference ID numbers 130, E37, and E38, Table S1). Perhaps because of the variability of phosphorus loss that spans organic and conventional systems and the inconsistency in which pathway is a primary contributor to pollution for that agricultural system, few policies specifically target phosphorus for water quality goals as they relate to the type of agricultural management.

The greater loss of carbon in runoff with organic treatments compared to conventional treatments was not surprising considering that organic agriculture is associated with techniques such as compost application that increase carbon on fields and in soil (Abdelrahman et al., 2020; Leifeld & Fuhrer, 2010). A prior meta‐analysis using 74 studies identified significantly greater soil organic carbon concentrations in organic agricultural fields than conventionally farmed fields in all comparisons as well as from a subset of comparisons where the practices used zero net inputs (Gattinger et al., 2012). So, both carbon sequestering practices such as using cover crops and adding carbon‐based fertilizers such as compost or leaving plant residue on the fields after harvest will boost the overall carbon content of the fields and therefore the amount susceptible to loss. The increase in carbon loss from organic systems was also one of the most variable factors, suggesting that without knowing the source of carbon and variation in the carbon species, it could be a poor target for water quality improvements through changing agricultural systems. In general, carbon inputs are not a priority concern for water quality aside from interactions with other chemicals during purification of drinking water that create disinfection by‐products and effects on aquatic organism trophic interactions (Brown, 2003). Dissolved and particulate carbon can also be associated with changes in water color that can affect light absorption and with higher turbidity, respectively (Gao & Zepp, 1998; Sehgal et al., 2022; Slaets et al., 2014; Thrane et al., 2014).

Over three‐quarters of the 25 paired comparisons that measured soil loss metrics identified an improvement with organic agriculture, but the mass difference in soil loss and measurement techniques was highly variable, limiting a clear expectation of the outcome from using an agricultural management type. This may be due in part to the strong influence that we found tillage practices to have on soil loss, irrespective of agricultural management type. We also found that organic management did not consistently reduce runoff. Prior studies show that a reduction in tillage can increase aggregate stability and infiltration, which can decrease runoff and particle loss (Barthès & Roose, 2002; DeLaune & Sij, 2012; Zhang et al., 2007). In our study, mechanisms for controlling weeds were often unspecified, but when reported, mechanical tilling was reported in conventional fields for 2% more studies than it was in organic fields. Both conventional and organic management practices can use a range of tillage intensities over the course of field preparation, planting, and weed control. Novel approaches in both conventional (or conservation) and organic management can decrease tillage intensities through no‐till planting or other methods such as weed control using electricity, roller crimping, fire, or in the case of conventional management, herbicides (Bond & Grundy, 2001; Frasconi et al., 2019; Kumar et al., 2020).

Soil pools of organic matter, nitrogen, and phosphorus vary with agricultural practices and affect the ability of a soil to retain nutrients (Bohoussou et al., 2023; Green et al., 2005). While this review did not find consistent evidence that soil nutrient content alone determines whether conventional or organic systems lose more nitrogen, phosphorus, carbon, or sediment, other studies suggest it can be an important contributing factor, particularly when soil nitrogen levels meet or exceed crop demand (Wang et al., 2019). For soil organic matter, there is little agreement across studies that organic agriculture boosts soil organic matter (Lorenz & Lal, 2022), unless the source of organic fertility is taken into account (Lorenz et al., 2025). Tillage practices can also be informative: systems that support soil aggregate development through, for example, limited tillage, were found to have greater soil carbon, nitrogen, and phosphorus in large aggregates and less susceptibility of those aggregates to particulate nutrient loss (Green et al., 2005).

This review highlights both the variation in methods of organic agricultural practices and the sparsity of studies using paired field comparisons to determine the effect of organic agricultural management practices on water quality. There is greater emphasis on the impact of farming systems on soil ecological quality that has led to over 100 publications per year by 2020, allowing for robust meta‐analysis comparing conventional and organic farming soil health (Christel et al., 2021). For water quality studies, we found that only three variables (nitrate leached, nitrogen leached, and runoff volume) had more than 10 paired comparisons, which severely limits the power of an analysis. There was also a low diversity of global locations and soil types that our search captured, with 76% of papers from European studies (primarily Denmark, Germany, and Italy) and 24% from North American studies (including eastern, western, and central), although within these continents, both more‐ and less‐intensive agricultural regions were included. Soil type was nearly 50% sandy loam. These limitations highlight opportunities for future research and evaluating definitions for global inclusivity that would strengthen our understanding of how different agricultural managements influence water quality. Further, they emphasize the potential for multivariate approaches such as partial least squares path modeling (Vinzi et al., 2010) or, as more data are available, machine learning (Benos et al., 2021) to identify the most influential individual or subsets of practices.

One focal point for future studies lies in legacy contaminants and contaminants of emerging concern including pesticides, veterinary pharmaceuticals, and per‐ and polyfluoroalkyl substances, which have been associated to the application of biosolids (e.g., Oviedo‐Vargas et al., 2025; Peter & Lee, 2025). We anticipated a major decline in the detection and concentrations of synthetic pesticides between conventionally farmed and organically farmed fields. Primarily, the pesticide detection and concentration were lower in water from the organic fields, but some pesticide detection did increase in organic field water. Previous studies of synthetic pesticide contamination of organic produce in Europe indicate that contamination is widespread and difficult to avoid, arising from routine sources and reflecting the widespread occurrence of pesticides in terrestrial and aquatic ecosystems (Schleiffer & Speiser, 2022). Our study cataloged 12 pesticides and highlights that more information is needed to help determine whether there are consistent effects on certain pesticides.

An essential piece of information in comparing agricultural managements is whether and for how long a field had been farmed conventionally before transitioning to organic practices, and the duration of the organic practices. As of 2021, in the United States, organic farmland was less than 1% of total farmland, but had more than doubled since 2000 to nearly 2 million ha (Carlson et al., 2023). For the studies we compared, the information was often available, but due to limited power could not be used to explain the variations in water quality metrics. However, these timescales could be integrated to better understand the effects of chemical legacies on water quality after conversion to organic agriculture.

Water quality parameters are but one set of variables in the equation that determines the environmental footprint of different agricultural management systems. To fully weigh the consequence of each system, differences in crop yields among management systems are a key consideration as this could change the total land area required for agriculture. One study, in Switzerland, identified lower yields with organic farming for wheat and corn and indicated that additional land would be required to meet the same production demands (Wittwer et al., 2021). Yield data from another study in the Mid‐Atlantic United States with rain‐fed fields indicated no difference in 3 years of yield from conventional and organically grown crops (Lotter et al., 2003). However, the following year of that study captured climate extremes in which crops experienced a drought followed by a September hurricane, and under these conditions, organically grown crops had higher yields. This highlights the importance of considering fluctuations in yield within and among agricultural systems under future climate scenarios. When production consistently differs by agricultural management, this could translate into unequal land areas needed for conventional and organic‐based agriculture to deliver equivalent row crop yields and change the calculation of gross water quality impacts. Tuomisto et al. (2012) demonstrated this in their meta‐analysis, which showed organic fields had lower nitrogen leaching per unit of area, but higher nitrogen leaching per production unit. Thus, framing water quality impacts as a unit of nitrate per agricultural production would be informative for thinking regionally and globally about the relationships between agriculture and water quality.

Several meta‐analyses have found that, in general, crops grown using organic agriculture have lower yields than those grown with conventional agriculture by around 18%–20% but with considerable variation (95% confidence interval, 77%–89%; standard deviation, 21%; and 95% confidence interval for each meta‐analysis, 16%–23%, respectively) attributed to crop type, climate, and soil type among other variables (de la Cruz et al., 2023; de Ponti et al., 2012; Ponisio et al., 2015). Given that more land may then be needed to meet food demands, are the benefits to water quality from organic agriculture cancelled out due to the greater area of land that is cultivated? In the case of nitrogen losses, Tuomisto et al.’s (2012) meta‐analysis suggests that, yes, the overall nitrogen leaching of organic agriculture would exceed that of conventional agriculture. A meta‐analysis by Mondelaers et al. (2009) also identified that for nitrate or phosphorus leaching, land use efficiency was an important consideration that could reduce or eliminate the benefits from organic systems. However, the case of soil loss requires considering the outcome over the long term. Soil erosion typically, though not exclusively, reduces land productivity (Lal & Moldenhauer, 1987), which in some soil types can be ameliorated by soil amendments and irrigation. In cases where amendments are unsuccessful, such as some tropical soils (Lal & Moldenhauer, 1987), the land may be abandoned and new arable tracts sought.

The use of pesticides and herbicides is perhaps the most notable difference between conventional and organic agriculture that influences water quality, irrespective of land area. Of the 17 pesticides or herbicides in our review, we identified only dicamba and bromoxynil in more water samples from organic treatments. These findings arose from one study and are not unheard of. In a systematic review by Sanders et al. (2025), there was a higher risk of pesticides from conventional farms in only three of 62 comparisons, and this was attributed to drift from adjacent farms. Certifying agencies differ in their requirements for production, but most pesticides (which are primarily synthetic) are prohibited for use in certified organic produce (e.g., United States Department of Agriculture [USDA] certification 7 eC.F.R. §205.105 2025) (Seufert et al., 2017; Speiser & Tamm, 2011). As many pesticides are severely detrimental to aquatic life and drinking water supplies, significant water quality improvements may stem from organic agricultural systems; however, the lack of data, legacy of historical pesticide use, and potential for adjacent organic farms to influence pesticide use on conventional farms poses a challenge to these analyses (Larsen et al., 2024; Liess et al., 2021; Saha & Dutta, 2024; Schleiffer & Speiser, 2022; Syafrudin et al., 2021).

The quantity of land to meet food production demands is not the only consideration, and water quality is one part of the larger environmental footprint. Similar to yield differences between organic and conventional systems, environmental and carbon footprints are highly variable and are challenging to measure consistently (Adewale et al., 2018; Meier et al., 2015). The duration of using the same agricultural system may also influence the sustainability outcome: in a study of a 23‐year‐old wheat–rice cropping system in India, the organic system had not only greater crop yields but also reduced water use and had a lower carbon footprint (Saikia et al., 2026). Boone et al. (2019) argue that including ecosystem services in the allocation for life cycle assessments (LCAs) shifts the calculation such that, under this scope, organic agriculture reduced resource consumption for corn, potatoes, and peas (*Pisum sativum* L.), but not for cereal grains. Regardless, an important consideration in LCA (and we would argue, also for water quality) is whether to calculate to unit of product or to unit of area as the former favors conventional agriculture, while the latter favors organic agriculture (Hashemi et al., 2024).

## 5 CONCLUSIONS

In this synthesis, we were able to identify patterns that nitrogen‐based and soil loss parameters more often improved with organic agriculture management, while phosphorus and carbon loss parameters more often worsened under organic management practices when compared with a paired conventionally managed row‐crop field. Our review highlights the importance of accounting for additional management practices such as no‐till, cover crops, or the type of organic fertilizer used to parse the mechanisms behind these varied water quality responses. Collectively, the scarcity of paired field studies also limits our understanding of how conventional or organic agriculture generally influences water quality parameters. Increasing the number of paired studies while also diversifying soil types, using area‐based metrics, and accounting for legacy management effects will help reduce uncertainty about how different agriculture managements will influence the quality of surface and groundwater.

## AUTHOR CONTRIBUTIONS

**Raven Bier**: Data curation; formal analysis; investigation; methodology; software; validation; visualization; writing—original draft; writing—review and editing. **Melinda Daniels**: Conceptualization; funding acquisition; methodology; project administration; resources; writing—original draft; writing—review and editing. **Diana Oviedo‐Vargas**: Conceptualization; funding acquisition; methodology; project administration; resources; writing—original draft; writing—review and editing. **Marc Peipoch**: Funding acquisition; project administration; writing—review and editing. **Emma Kelsick**: Data curation; validation; writing—review and editing. **Jinjun Kan**: Conceptualization; funding acquisition; investigation; project administration; resources; supervision; writing—original draft; writing—review and editing.

## CONFLICT OF INTEREST STATEMENT

The authors declare no conflicts of interest.

## Supporting information



Supporting information

Supplemental Table S1 contains the literature studies used in this review as referenced by ID number.

Supplemental Table S2 provides the characteristics of the field studies included in this review.

Supplemental Table S3 provides the ranges, means, and medians of conventional and organic agriculture treatment values.

## Data Availability

Readers can find and access the data from published papers in Supplemental Table S1 and also by contacting the corresponding author. No new data were collected in this review. Published data are available from literature references provided in Supplemental Table S1.
