# Supplementary material for: Surface water quality impacts from organic versus conventional agricultural systems
Source: J Environ Qual. 2026 Jun 30;55(4):e70208. doi: 10.1002/jeq2.70208 (PMC13316142; doi:10.1002/jeq2.70208)
Supplement: Supplementary file 1 — Supporting information [file JEQ2-55-0-s001.docx]

Supplemental Information for:

**Surface water quality impacts from organic versus conventional agricultural systems**

Raven Bier^1*^, Melinda Daniels^2^, Diana Oviedo-Vargas^2^, Marc Peipoch^2^, Emma Kelsick^1,3^, and Jinjun Kan^2^

^1^ Savannah River Ecology Laboratory, University of Georgia, Aiken, South Carolina 29802, USA

^2^ Stroud Water Research Center, 970 Spencer Road, Avondale, Pennsylvania 19311, USA

^3^ Odum School of Ecology, University of Georgia, Athens, Georgia 30602, USA

* Correspondence to: Raven Bier (rbier@srel.uga.edu)

Content:

Supplemental Table S1 (.xlsx file)

Supplemental Table S2 (.xlsx file)

Supplemental Table S3 (.xlsx file)
